# More on the first-order thermodynamics of scalar-tensor and Horndeski gravity

**DOI:** 10.1140/epjc/s10052-023-11712-7

**Published:** 2023-06-20

**Authors:** Valerio Faraoni, Julien Houle

**Affiliations:** Department of Physics and Astronomy, Bishop’s University, 2600 College Street, Sherbrooke, QC J1M 1Z7 Canada

## Abstract

Two issues in the first-order thermodynamics of scalar-tensor (including “viable” Horndeski) gravity are elucidated. The application of this new formalism to FLRW cosmology is shown to be fully legitimate and then extended to all Bianchi universes. It is shown that the formalism holds thanks to the almost miraculous fact that the constitutive relations of Eckart’s thermodynamics are satisfied, while writing the field equations as effective Einstein equations with an effective dissipative fluid does not contain new physics.

## Introduction

There are many motivations to consider seriously theories of gravity alternative to General Relativity (GR) [1]. Attempts to quantum-correct GR generally lead to new degrees of freedom (in addition to the two familiar massless spin two modes), higher order equations of motion, extra fields, or non-local features. The low-energy limit of the bosonic string, the simplest string theory, does not reproduce Einstein gravity but gives an $\omega =-1$ Brans–Dicke theory instead (where $\omega$ is the Brans–Dicke parameter) [2, 3]. More compelling motivation comes from the accelerated expansion of the universe: explaining the present-day cosmic acceleration within the context of GR requires the introduction of a completely ad hoc dark energy, the nature of which remains mysterious [4].

The simplest alternative to GR is scalar-tensor gravity, which adds only a scalar degree of freedom $\phi$ to the two degrees of freedom contained in the metric $g_{ab}$ in GR. A subclass of scalar-tensor gravity, *f*(*R*) theories, seems to be the most popular alternative to GR to explain the cosmic acceleration ([5, 6], see [7–9] for reviews). During the past decade Horndeski gravity [10] was rediscovered while attempting to write down the most general scalar-tensor theory with second-order equations of motion. Although this record ultimately belongs to the newly-discovered Degenerate Higher Order Scalar-Tensor (DHOST) theories more general than Horndeski’s, the study of Horndeski gravity has flourished, generating a large literature (see, e.g., [11–26, 28–35] for its various aspects, including cosmology, and for reviews, and see [36] for constraints on DHOST from solar physics).

At the same time, the idea that gravity may be different from the other three fundamental forces and may be emergent instead, has taken a firm foot in the literature in various forms (see [37–44] for reviews). A particularly deep approach is Jacobson’s thermodynamics of spacetime, in which the Einstein equation is derived from thermodynamics [45]. When applied to (metric) *f*(*R*) gravity [46], it embodies the idea that GR constitutes a state of equilibrium for gravity while *f*(*R*) gravity is an out-of equilibrium state ( [46], see also [47]). This idea has been adopted in the completely different first-order thermodynamics of scalar-tensor gravity recently proposed [48–51, 53]. This new formalism begins from the realization that the field equations of “first-generation” scalar-tensor [54–60] and Horndeski gravity can be rewritten in the form of effective Einstein equations (using the notations of Ref. [61])

1 $$\begin{aligned} R_{ab}-\frac{1}{2} \, g_{ab} R = 8\pi \left( T_{ab}^{(\phi )} + \frac{ T_{ab}^\mathrm {(matter)} }{\phi } \right) , \end{aligned}$$

where $$R_{ab}$$ is the Ricci tensor of the metric $$ g_{ab}$$ and $$T_{ab}^\mathrm {(matter)}$$ is the matter stress-energy tensor, while $$T_{ab}^{(\phi )}$$ is an effective stress-energy tensor built out of the gravitational scalar $$\phi $$ and its first and second covariant derivatives (indeed, this is the usual way to present “first generation” scalar-tensor gravity). Furthermore, the effective $$T_{ab}^{(\phi )}$$ assumes the form of an imperfect fluid energy-momentum tensor

2 $$\begin{aligned} T_{ab}= \rho u_a u_b +P h_{ab} +\pi _{ab} + q_a u_b + q_b u_a , \end{aligned}$$

where $$ u^a$$ is the fluid 4-velocity (normalized to $$u_c u^c=-1$$), $$h_{ab} \equiv g_{ab}+u_a u_b$$ is the metric of the 3-space seen by the observers with 4-velocity $$u^a$$ comoving with the fluid, $$\rho $$ is the energy density, *P* the isotropic pressure, $$\pi _{ab}$$ is the anisotropic stress tensor, and $$q^a$$ is a heat flux density. $$P = {\bar{P}}+P_\textrm{viscous}$$ is the sum of a perfect fluid contribution $${\bar{P}}$$ and of a viscous pressure; $$h_{ab}, \pi _{ab}$$, and $$q^a$$ are purely spatial,

3 $$\begin{aligned} h_{ab} u^a=h_{ab}u^b=q_a u^a =\pi _{ab} u^a =\pi _{ab}u^b=0 , \end{aligned}$$

and $\pi _{ab}$ is trace-free, ${\pi _a}^a=0$.

The dissipative fluid nature of the effective scalar field stress-energy tensor was recognized in [48, 62] for “old” scalar-tensor gravity and in [51, 63] for Horndeski gravity. In fact, *any* symmetric two-index tensor can be decomposed in the form (2) (more on this in Sect. 4). What makes the analogy between scalar field and dissipative fluid meaningful is the fact that the effective fluid quantities satisfy the constitutive relations of Eckart’s first-order thermodynamics [64]

4 $$\begin{aligned}{} & {} q_a = - K h_{ab} \left( \nabla ^b {{\mathscr {T}}} + {{\mathscr {T}}} {\dot{u}}^b \right) , \end{aligned}$$

5 $$\begin{aligned}{} & {} \pi _{ab} = -2\eta \, \sigma _{ab} ,\end{aligned}$$

6 $$\begin{aligned}{} & {} P_\textrm{viscous} = -\zeta \, \Theta , \end{aligned}$$

where ${{\mathscr {T}}}$ is the temperature, *K* is the thermal conductivity, and $\Theta , \sigma _{ab}$ are the expansion and trace-free shear of the 4-velocity $u^a$, while ${\dot{u}} \equiv u^c\nabla _c u^a$ is the fluid 4-acceleration. The fact that the *effective* $T_{ab}^{(\phi )}$ satisfies Eqs. (4)–(6) was realized for general “old” scalar-tensor gravity in [48] (and, for particular geometries or theories, in previous references [65]) and in “viable” Horndeski gravity in [51, 63] and identifies a “temperature of gravity” with respect to GR. Einstein gravity, recovered for $\phi =$ const., corresponds to zero temperature while scalar-tensor gravity is an excited state. This idea is plausible: if the field content of gravity consists of the two massless spin two modes of GR plus a propagating scalar mode, exciting the latter corresponds to an excited state with respect to GR. The whole idea of the first-order thermodynamics of scalar-tensor gravity consists of taking seriously the dissipative form of the effective $T_{ab}^{(\phi )}$ and applying Eckart’s thermodynamics to it. It is something akin to a miracle that Eckart’s constitutive relations are satisfied [48, 49, 51]. With all the limitations intrinsic to Eckart’s thermodynamics (lack of causality and instabilities [66, 67]), an intriguing thermal picture of modified gravity emerges [48–51, 53], which is under development. Ideas and tools partially or fully developed include: an explicit equation describing the approach to the GR equilibrium or the departures from it; the expansion of space causes the “cooling” of gravity; near spacetime singularities and singularities of the effective gravitational coupling, where the scalar degree of freedom is fully excited, gravity is “hot” and deviates radically from GR; in cosmology only bulk viscosity survives due to the spacetime symmetries; states of equilibrium corresponding to $K{{\mathscr {T}}}=0$ (or even $K {{\mathscr {T}}}=$ const.) distinct from GR can exist, corresponding to non-dynamical scalar fields or to metastable states [68–70].^1^

We summarize Horndeski theory for use in the following sections. Denoting $X \equiv -\frac{1}{2} \, \nabla ^c \phi \nabla _c \phi$, the Lagrangian of Horndeski gravity reads

7 $$\begin{aligned} \mathscr {L} = \mathscr {L}_2 + \mathscr {L}_3 + \mathscr {L}_4 + \mathscr {L}_5 , \end{aligned}$$

where

8 $$\begin{aligned} \mathscr {L}_2= & {} G_2 \left( \phi , X \right) \,, \end{aligned}$$

9 $$\begin{aligned} \mathscr {L}_3= & {} - G_3\left( \phi , X \right) \, \Box \phi \,,\end{aligned}$$

10 $$\begin{aligned} \mathscr {L}_4= & {} G_4\left( \phi , X \right) \, R + G_{4 X}\left( \phi , X \right) \left[ (\Box \phi )^2 - (\nabla _a \nabla _b \phi )^2 \right] , \nonumber \\ \end{aligned}$$

11 $$\begin{aligned} \mathscr {L}_5= & {} G_5\left( \phi , X \right) \, G_{ab} \, \nabla ^a \nabla ^b \phi - \frac{G_{5X}\left( \phi , X \right) }{6} \nonumber \\{} & {} \times \Big [ (\Box \phi )^3 - 3 \, \Box \phi \, (\nabla _a \nabla _b \phi )^2 + 2 \, (\nabla _a \nabla _b \phi )^3 \Big ] , \end{aligned}$$

and where $\nabla _a$ is the covariant derivative of $g_{ab}$, $\Box \equiv g^{ab} \nabla _a \nabla _b$, $G_{ab}$ is the Einstein tensor, while $G_i (\phi , X)$ ($i=2,3,4,5$) are arbitrary functions of $\phi$ and *X*, while $G_{i\phi } \equiv \partial G_i / \partial \phi$,  $G_{iX} \equiv \partial G_i / \partial X$.

Horndeski gravity is constrained theoretically by the need to avoid graviton decay into scalar field perturbations [25] and, above all, by the 2017 multi-messenger observation of gravitational waves and $\gamma$-rays emitted simultaneously in the GW170817/GRB170817 event [71, 72], which sets a stringent upper limit on the difference between the propagation speeds of gravitational and electromagnetic waves [73]. The subclass of Horndeski theories that implies luminal propagation of gravitational waves has $G_{4 X} = 0$, $G_5=0$ and its Lagrangian density is restricted to

12 $$\begin{aligned} \bar{{\mathscr {L}}}= G_2 (\phi , X) - G_3 (\phi , X) \Box \phi + G_4 (\phi ) R . \end{aligned}$$

This is not the only constraint on viable Horndeski gravity. Further constraints on the viable class as a replacement of the ad hoc dark energy come from attempts to address the Hubble tension on the present value $H_0$ of the Hubble parameter. An Effective Field Theory description of dark energy with scalar fields seems unable to accommodate $H_0$ and baryon acoustic oscillations [27]. In the following we proceed with the Lagrangian density (12).

## Correct generalization of Fourier’s law

Eckart’s generalization of Fourier’s law [64] is often reported as

13 $$\begin{aligned} q_a =- K \left( h_{ab} \nabla ^b {{\mathscr {T}}} + {{\mathscr {T}}} {\dot{u}}_a \right) . \end{aligned}$$

The heat flux density $q^a$ is purely spatial in Eckart’s theory and, therefore, non-causal, an unphysical feature corrected in the Israel-Stewart second-order thermodynamics and in later formalisms. While, in the right-hand side of Eq. (13), $- K h_{ab} \nabla ^b {{\mathscr {T}}}$ is trivially a purely spatial vector (it is a projection onto the 3-space orthogonal to $u^a$), the second term $- K {{\mathscr {T}}} {\dot{u}}^a$ proportional to the fluid 4-acceleration is not always a spatial vector, contrary to intuition. While most of the times a particle’s 4-acceleration is orthogonal to the particle 4-velocity, this is not always the case. Although at first sight this may seem hair-splitting, relevant situations discussed in the literature span a range of interesting subjects including particles with varying mass, the Einstein frame of scalar-tensor gravity, cosmic antifriction due to self-interacting dark matter or to particle production, and Friedmann–Lemaître–Robertson–Walker (FLRW) cosmology sourced by a perfect fluid with pressure in the comoving frame [74]. There is an abundant literature on analytic solutions of the Einstein equations describing mass-varying systems such as rockets and solar sails in GR (e.g., [75–77] and references therein) and mass-changing particles in cosmology and in scalar-tensor gravity [78–83]. In the early universe, quantum processes can create particles, a phenomenon associated with negative bulk pressures [84, 85], and it has been suggested that such a mechanism could drive inflation [86–91]. Negative bulk stresses can be caused by the self-interaction of dark matter, which has been investigated as a possible cause of the present cosmic acceleration [86] because it causes a cosmic “antifriction” on the dark matter fluid, a force antiparallel to the worldlines of dark matter particles [86].

In the Einstein conformal frame of scalar-tensor cosmology, a similar 4-force parallel to the trajectory appears. It can be interpreted as due to the fact that what was the constant mass of a test particle in the Jordan frame now depends on the Brans–Dicke-like scalar $\phi$ (that is, upon transformation to the Einstein conformal frame massive test particles cease being test particles and are subject to a fifth force proportional to $\nabla ^a\phi$) [74, 92]. In the low-energy limit of string theories, the geodesic equation of dilaton gravity contains a similar correction but, in general, the coupling of the dilaton to particles of the Standard Model is not universal [93–95].

Consider a FLRW universe with line element

14 $$\begin{aligned} ds^2 =-dt^2 +a^2(t) \left[ \frac{dr^2}{1-kr^2} +r^2 \left( d\vartheta ^2 +\sin ^2\vartheta \, d\varphi ^2 \right) \right] \nonumber \\ \end{aligned}$$

in comoving coordinates $\left( t, r, \vartheta , \varphi \right)$, that is, in the frame adapted to the symmetries (spatial homogeneity and isotropy) and comoving with the perfect fluid usually causing gravity. Unless this fluid is a dust or the effective fluid $T_{ab}^{(\Lambda )}=-\frac{\Lambda }{8\pi } \, g_{ab}$ describing a cosmological constant term with constant pressure, there are a pressure *P*(*t*) and a pressure gradient $\nabla _a P \ne 0$, hence a 4-force pointing in the time direction $u^a$. The presence of this force makes fluid particles deviate from geodesics, hence they have a 4-acceleration. This is easy to understand since, due to the symmetries, this 4-acceleration and 4-force cannot have spatial components in the comoving frame. As a result, the (massive) fluid particles satisfy the equation of motion [74]

15 $$\begin{aligned} \frac{d^2 x^{\mu }}{dt^2} + \Gamma ^{\mu }_{\alpha \beta } \, \frac{dx^{\alpha }}{dt} \, \frac{ dx^{\beta }}{dt} = A \, \frac{dx^{\mu }}{dt} , \end{aligned}$$

where *A* is a function of the position on the timelike trajectory. Equation (15) is recognized as the non-affinely parameterized timelike geodesic equation with the comoving time *t* coinciding with the the proper time of comoving observers. It is always possible to change parametrization to an affine parameter in which the right-hand side of the geodesic equation vanishes, hence this 4-acceleration is regarded as trivial and usually described as vanishing, but the reparametrization entails the use of an affine parameter that is not the comoving time *t*, which is the proper time of comoving observers. (If *s* is an affine parameter, the function *A* in Eq. (15) is $A(t)= \frac{dt}{ds} \, \frac{d^2s}{dt^2}$ [74, 96], see Appendix A.) In other words, the equation describing the spacetime trajectory of the fluid particles cannot be affinely parametrized by the proper time of comoving observers and there is a 4-force parallel to the 4-velocity $u^a$ in this frame [74] (see Appendix A).^2^ While this is immaterial for the mathematics of curves, the difference between proper time of comoving observers and another parameter is important for the physics because the description of FLRW cosmology is always done with respect to comoving observers who see the cosmic microwave background homogeneous and isotropic around them (apart from tiny temperature fluctuations).

It is clear then that, in Eckart’s first constitutive relation (13), the term $- K{{\mathscr {T}}} {\dot{u}}_a$ contributing to the heat flux density $q_a$ is not always purely spatial and, as a result, $q_a$ is not purely spatial, either. This fact is important because the effective first-order thermodynamics of scalar-tensor and Horndeski gravity à la Eckart, including FLRW cosmologies, is based on Eckart’s generalization of Fourier’s law. It is easy to fix Eq. (13) to make the heat flux density $q_a$ purely spatial in all situations: it is sufficient to write^3^

16 $$\begin{aligned} q_a = - K h_{ab} \left( \nabla ^b {{\mathscr {T}}}+{{\mathscr {T}}} {\dot{u}}^a \right) , \end{aligned}$$

i.e., projecting both temperature gradient $\nabla ^b {{\mathscr {T}}}$ and the 4-acceleration of the dissipative fluid onto the 3-space orthogonal to $u^a$.

An apparent puzzle remains in the study of the first-order thermodynamics of scalar-tensor and Horndeski gravity in FLRW cosmology, which is addressed in the next section.

## Scalar-tensor thermodynamics in FLRW and in Bianchi cosmology

The study of Eckart’s thermodynamics in FLRW cosmology has been carried out for first-generation scalar-tensor gravity [53] and is being generalized to spatially anisotropic Bianchi cosmologies and to Horndeski gravity. To put these studies on a firm footing, we elucidate the validity of its formulas involving the effective temperature in cosmology, where the heat flux $q^a$ vanishes identically.

Let $\phi$ be the gravitational scalar degree of freedom of the theory and $X \equiv -\frac{1}{2} \, \nabla ^c\phi \nabla _c \phi$. In general, scalar-tensor thermodynamics is studied in the comoving frame, i.e., the frame moving with the effective fluid 4-velocity, in which the effective fluid is at rest (this is natural in tensor-single-scalar gravity; the analogue of the comoving frame becomes artificial in tensor-multi-scalar gravity [97]). Applying this formalism to FLRW cosmology, it is clear that the purely spatial heat flux density (16) must vanish in the comoving frame to respect the FLRW symmetries. However, the fundamental relation of this thermodynamics

17 $$\begin{aligned} K{{\mathscr {T}}} = \frac{\sqrt{2X}}{8\pi \phi } \end{aligned}$$

in “first generation” scalar-tensor gravity, or its counterpart

18 $$\begin{aligned} K{{\mathscr {T}}} = \frac{\sqrt{2X} \, \left( G_{4\phi } -XG_{3X} \right) }{G_4} \end{aligned}$$

in viable Horndeski gravity, are derived from identifying the effective heat flux density $q_a$ of Eq. (16) in these theories with $- K {{\mathscr {T}}} h_{ab} {\dot{u}}^b$. The relation (17) or (18) derived in the general theory still holds in FLRW cosmology. $q_a$ vanishes identically in the comoving frame not because $K{{\mathscr {T}}}=0$ but *because* $h_{ab}{\dot{u}}^b =0$ *in any FLRW universe*.

Let us be more specific: in Horndeski gravity, the 4-velocity of the effective fluid is

19 $$\begin{aligned} u^a = \frac{\nabla ^a \phi }{\sqrt{2X}} \end{aligned}$$

(the analogy is meaningful if $\nabla ^a\phi$ is timelike and future-oriented [98]) and the fluid’s 4-acceleration turns out to be

20 $$\begin{aligned} {\dot{u}}^a \equiv u^c \nabla _c u^a = -\frac{1}{2X} \left( \nabla ^a X + \frac{ \nabla ^c X \nabla _c \phi }{2X} \, \nabla ^a \phi \right) . \end{aligned}$$

Its projection onto the 3-space orthogonal to $$u^a $$ vanishes if and only if $${\dot{u}}^a = \alpha \, u^a$$ (where $$\alpha $$ is a function of the spacetime coordinates), or

21 $$\begin{aligned} \nabla ^a X + \frac{ {\dot{X}}}{\sqrt{2X}} \, \nabla ^a \phi =-2\alpha X u^a \end{aligned}$$

(where $${\dot{X}} \equiv u^c \nabla _c X$$), or

22 $$\begin{aligned} \nabla ^a X =-\left( {\dot{X}} + 2\alpha X \right) u^a , \end{aligned}$$

i.e., if $\nabla ^a X$ is parallel to the effective fluid 4-velocity. In a FLRW universe, or in any space in which $g^{00}$ depends only on the comoving time *t* and $\phi =\phi (t)$ we have, in comoving coordinates,

23 $$\begin{aligned} \nabla _a X = \partial _a X = - \frac{\dot{\phi }}{2} \left( \dot{\phi } \, \partial _t g^{00} + 2g^{00} \ddot{\phi } \right) {\delta _a}^0 . \end{aligned}$$

Using $u_a=\frac{\nabla _a \phi }{\sqrt{2X}} = \frac{\dot{\phi }}{\sqrt{2X}} \, {\delta _a}^0$, one writes

24 $$\begin{aligned} \nabla _a X = - \sqrt{ \frac{X}{2}} \, \left( \dot{\phi } \, \partial _t g^{00} + 2 g^{00} \, \ddot{\phi } \right) u_a . \nonumber \\ \end{aligned}$$

In the FLRW geometry written in comoving coordinates it is $g^{00}=-1$ and $\nabla _a X$ reduces to $- |\dot{\phi }| \ddot{\phi } \, u_a$, which is indeed parallel to $u^a$ and then $h_{ab} \, {\dot{u}}^b =0$. The heat flux density of viable Horndeski gravity [51]

25 $$\begin{aligned} q_a= \sqrt{2X} \, \frac{ \left( G_{4\phi }-XG_{3X} \right) }{G_4} \, h_{ab} {\dot{u}}^b =- K{{\mathscr {T}}} h_{ab} {\dot{u}}^b \end{aligned}$$

vanishes not because $K{{\mathscr {T}}}=0$ but because $h_{ab} {\dot{u}}^b=0$ (even though ${\dot{u}}^b$ is non-vanishing in FLRW cosmology).

The same situation occurs in Bianchi universes in which, again, $g^{00}=-1$ in comoving coordinates. Consider first vacuum Horndeski gravity, in which the $\phi$-fluid is the only source in the effective Einstein equations. The 4-velocity of this effective fluid comes from a gradient, therefore it has zero vorticity, $\omega _{ab}=0$. Then the Frobenius theorem guarantees that $u^a$ is hypersurface-orthogonal and excludes the possibility that the $\phi$-fluid is tilted with respect to the Bianchi observers (the observers that see the 3-space of a Bianchi universe as homogeneous) [61, 96]. There is a time *t* (“comoving time”) such that the 3-surfaces $t=$ const. are spacelike surfaces of homogeneity, $\phi =\phi (t)$, and $u^a$ coincides with the unit normal to these hypersurfaces. Denoting with $x^i$ ($i=1,2,3$) the spatial coordinates on these $t=$ const. hypersurfaces, the Bianchi line element in comoving coordinates is [96, 99, 100]

26 $$\begin{aligned} ds^2=-dt^2 + \gamma _{ij}(t) \, \text{ e}^{\beta _i(t)} \, dx^i dx^j \quad \quad (i,j=1,2,3) \end{aligned}$$

with $$g_{00}=-1 $$, $$g_{0i}=0$$ (that is, comoving and synchronous coordinates coincide), and where $$ \sigma _{ij}=\sigma _{ij}(t) $$. Equation (23) then gives again that $$\nabla _a X$$ is parallel to $$u_a$$, $$h_{ab} {\dot{u}}^b=0$$, and $$q_a=0$$ (moreover, in covariant notation, $$h_{ab} \nabla ^b P= h_{ab}\nabla ^b \sigma _{cd}=0$$).

Let us consider explicitly Bianchi I universes for illustration. Bianchi I models sourced by a single anisotropic fluid have line element [96, 99–101]

27 $$\begin{aligned} ds^2 =- dt^2 + a^2(t) \, \text{ e}^{ 2\beta _{(i)}(t) } \delta _{ij} dx^i dx^j \quad \quad (i,j=1,2,3)\nonumber \\ \end{aligned}$$

in comoving coordinates. If the single fluid sourcing the Bianchi I universe is the effective Horndeski $\phi$-fluid (i.e., in vacuum Horndeski cosmology), using Eq. (23) we find again ${\dot{u}}^b$ parallel to $u^b$ and $q_a=0$. A direct computation gives $q_a=0$ (e.g., [96, 100, 101]). This result still holds in the presence of a real fluid if its 4-velocity is aligned with $u^a$.

Let us consider now Horndeski gravity in the presence of matter, which is usually taken to be a fluid. If this fluid is not tilted with respect to the Horndeski effective fluid, then its 4-velocity coincides with $u^a$ given by Eq. (22) and the previous arguments apply again. This is not the case, in general, if the real fluid is tilted with respect to the effective one.

The discussion of this section legitimates the study of FLRW and Bianchi cosmology in the first-order thermodynamics of scalar-tensor gravity. These discussions draw conclusions based on $K{{\mathscr {T}}}$ [53] even though the heat flux vector (13) (from which $K{{\mathscr {T}}}$ is derived) vanishes identically in the comoving frame of the Horndeski effective fluid.^4^ The reason why $q^a$ vanishes is not because $K {{\mathscr {T}}}$ is zero (which would invalidate the discussions of Eckart’s thermodynamics in cosmology), but because ${\dot{u}}^a$ is parallel to the trajectories of fluid particles (i.e., to $u^a$). (It is possible that $K{{\mathscr {T}}}$ is always zero in a certain specific Horndeski theory because $G_{4\phi }-XG_{3X}$ in Eq. (18) vanishes identically there, which makes this theory with non-dynamical $\phi$ a state of equilibrium alternative to GR [102]).

## Decomposition of any symmetric tensor in the “imperfect fluid” form

In order to appreciate the first-order thermodynamics of scalar-tensor or viable Horndeski gravity [48–53, 68–70, 97, 98], one should understand what is peculiar to the effective stress-energy tensor of these theories, once their field equations are written as effective Einstein equations. It is not the fact that their effective stress-energy tensor assumes the form of a dissipative fluid—this is true for *any* symmetric 2-index tensor. What is peculiar is the fact that this effective stress-energy tensor *satisfies the constitutive relations of Eckart’s first-order thermodynamics*. This property is truly remarkable and is certainly not warranted.^5^ Let us discuss explicitly the dissipative fluid decomposition of a symmetric tensor.

Given a timelike vector field $u^a$ normalized so that $u_c u^c=-1$, *any* symmetric 2-index tensor $S_{ab}=S_{ba}$ can be decomposed in the imperfect fluid form

28 $$\begin{aligned} S_{ab}=\rho u_a u_b +P h_{ab} + q_a u_b + q_b u_a + \pi _{ab} , \end{aligned}$$

where $h_{ab} \equiv g_{ab}+u_a u_b$ and $q^a$ and $\pi ^{ab}$ are purely spatial with respect to $u^a$, with $\pi ^{ab}$ symmetric and trace-free. This “imperfect fluid decomposition” is purely formal since, in general, the symmetric tensor $S_{ab}$ is not a real or effective stress-energy tensor, and does not even have the dimensions of stress-energy.

In general, the constitutive relations of Eckart’s first-order thermodynamics (4)–(6) are not satisfied by the components of $S_{ab}$, and neither is any other prescribed constitutive relation. By contrast, the effective stress-energy tensor of scalar-tensor gravity $T_{ab}^{(\phi )}$ satisfies Eckart’s constitutive relations, as does that of a restricted class of Horndeski theories of gravity [51, 102]. In general, given an alternative theory of gravity in vacuo, one can rewrite its field equations as effective Einstein equations with a suitable, symmetric, effective stress-energy tensor $T_{ab}^\mathrm {(eff)}$. However:

1. In general, a preferred 4-velocity vector field $u^a$ is not defined. If it is defined, as in scalar-tensor or Horndeski gravity where there is a scalar field $\phi$ and $\nabla ^a \phi$ singles out a preferred vector field, the fluid-dynamical analogy requires that
  $$\nabla ^c \phi $$ is timelike, $$\nabla ^c\phi \nabla _c \phi <0$$;
  $\nabla ^c\phi$ is future-oriented, $g_{ab} \nabla ^a \left( \partial _t \, \right) ^b <0$.
2. If a preferred (timelike, normalized, and future-oriented) vector field is not present in the alternative theory of gravity, one could choose one arbitrarily, which corresponds to choosing a family of physical observers in spacetime. Then, provided that the field equations of this theory can be written as effective Einstein equations, one has an effective symmetric $T_{ab}^\mathrm {(eff)}$ which can be decomposed in the form of an imperfect fluid. However, $u^c$ has no relation with the gravitational degrees of freedom of the theory and, in general, no constitutive relation is satisfied. This is intuitive: constitutive relations express the physical properties of a material (specifically, its response to mechanical and thermal stresses) and there is no physics in the purely geometric decomposition of a tensor into its temporal, spatial, and mixed components. It is remarkable that scalar-tensor gravity does indeed satisfy Eckart’s constitutive relations.

### Decomposition

Let $$S_{ab} $$ be any symmetric 2-index tensor in a spacetime endowed with a metric $$g_{ab}$$ and let $$u^a$$ be a timelike vector field. Without loss of generality, we can assume that $$u^a$$ is normalized to $$u_c u^c=-1$$ (otherwise one can always normalize it). Define the 3-metric $$ h_{ab} \equiv u_a u_b + g_{ab} $$  ($${h_a}^b$$ is the projector onto the 3-space seen by $$u^a$$, i.e., $$h_{ab}u^a =h_{ab} u^b=0$$). Then is is always possible to decompose $$S_{ab}$$ according to

29 $$\begin{aligned} S_{ab} = \rho u_a u_b + P h_{ab} + q_a u_b + q_b u_a +\pi _{ab} , \end{aligned}$$

where

30 $$\begin{aligned} q_a u^a =0 , \quad \quad \pi _{ab} u^a = \pi _{ab} u^b =0 , \quad \quad {\pi ^a}_a=0 . \end{aligned}$$

The quantities appearing in this decomposition are

31 $$\begin{aligned} \rho= & {} S_{ab} u^a u^b , \end{aligned}$$

32 $$\begin{aligned} P= & {} \frac{1}{3} \, h^{ab} S_{ab} ,\end{aligned}$$

33 $$\begin{aligned} q^a= & {} - {h^a}^c S_{cd} u^d ,\end{aligned}$$

34 $$\begin{aligned} \pi _{ab}= & {} \left( {h_a}^c \, {h_b}^d -\frac{1}{3} \, h_{ab} \, h^{cd} \right) S_{cd} . \end{aligned}$$

They are just the projections of $S_{ab}$ onto the time direction (projected twice for $\rho$), onto the 3-space (projected twice for the isotropic and anisotropic stresses $P h_{ab}$ and $\pi _{ab}$), and projected once onto the 3-space/once onto the time direction (for $q^a$). In this sense, the decomposition is rather obvious (it is mentioned, e.g., in [96, 103], but seems to have been missed by many authors discussing various scalar-tensor theories over the years).

*Proof.* By definition, $q^a$ and $\pi ^{ab}$ are purely spatial since they are a projection and a double projection onto the 3-space seen by $u^a$:

$$\begin{aligned} q_a u^a \equiv - {h_a}^c \left( S_{cd} u^d \right) u^a =0 \end{aligned}$$

and

$$\begin{aligned} \pi _{ab} u^a\equiv & {} \left( {h_a}^c \, {h_b}^d -\frac{1}{3} \, h_{ab} \, h^{cd} \right) S_{cd} u^a \\= & {} -\frac{1}{3} \, \left( h^{cd} S_{cd} \right) h_{ab} u^a=0 ,\\ \pi _{ab} u^b\equiv & {} \left( {h_a}^c \, {h_b}^d -\frac{1}{3} \, h_{ab} \, h^{cd} \right) S_{cd} u^b \\= & {} -\frac{1}{3} \, \left( h^{cd} S_{cd} \right) h_{ab} u^b=0 , \end{aligned}$$

and

$$\begin{aligned} {\pi ^a}_a = \left( {h_a}^c \, h^{ad} -\frac{h}{3} \, h^{cd} \right) S_{cd} =\left( h^{cd} -\frac{3}{3} \, h^{cd} \right) S_{cd}=0 . \end{aligned}$$

It is easy to show that, using the quantities (31)–(34), the right-hand side of Eq. (29) reproduces the given tensor $S_{ab}$. In fact,

$$\begin{aligned}{} & {} \rho u_a u_b + P h_{ab} + q_a u_b + q_b u_a +\pi _{ab} \equiv \left( S_{cd} u^c u^d \right) u_a u_b \\{} & {} \qquad + \left( \frac{ S_{cd} h^{cd}}{3} \right) h_{ab} - \left( {h_a}^c S_{cd} u^d \right) u_b \\{} & {} \qquad - \left( {h_b}^c S_{cd} u^d \right) u_a + \left( {h_a}^c \, {h_b}^d -\frac{ h^{cd} S_{cd}}{3} \right) S_{cd} \\{} & {} \quad = \left( S_{cd} u^c u^d \right) u_a u_b {+} \left( \frac{ S_{cd} h^{cd}}{3} \right) h_{ab} {-} \left( {\delta _a}^c {+} u_a u^c \right) \left( S_{cd} u^d \right) u_b \\{} & {} \qquad {-} \left( {\delta _b}^c {+} u_b u^c \right) \left( S_{cd} u^d \right) u_a {+} \left( {\delta _a}^c {+} u_a u^c \right) \left( {\delta _b}^d {+} u_b u^d \right) S_{cd} \\{} & {} \qquad -\frac{h_{ab}}{3} \left( g^{cd} + u^c u^d \right) S_{cd} \\{} & {} \quad = \left( S_{cd} u^c u^d \right) u_a u_b + \left( \frac{ S_{cd} h^{cd}}{3} \right) h_{ab} -\left( S_{ad} u^d \right) u_b \\{} & {} \qquad -\left( S_{cd} u^c u^d \right) u_a u_b -\left( S_{bd} u^d \right) u_a \\{} & {} \qquad -\left( S_{cd} u^c u^d \right) u_a u_b +S_{ab} +\left( S_{ad} u^d \right) u_b + \left( S_{cb} u^c \right) u_a \\{} & {} \qquad + \left( S_{cd} u^c u^d \right) u_a u_b -\frac{S}{3} \, h_{ab} - \frac{S_{cd} u^c u^d}{3} \, h_{ab} \\{} & {} \quad = S_{ab} +\frac{h_{ab}}{3} \left[ S_{cd}\left( g^{cd} +u^c u^d \right) -S -S_{cd} u^c u^d \right] \\{} & {} \quad = S_{ab} . \end{aligned}$$

### Effective constitutive relations

Apart from the fact that they do not have the dimensions of fluid quantities, in general the quantities appearing in the effective dissipative fluid decomposition do not satisfy effective constitutive relations. For example, the first of Eckart’s constitutive relations (4) corresponds to

35 $$\begin{aligned} S_{0i} = - K h_{ij} \left( \partial ^j {{\mathscr {T}}} +{{\mathscr {T}}} {\dot{u}}^j \right) \end{aligned}$$

and one cannot see how functions *K* and ${{\mathscr {T}}}$ could exist to satisfy this relation between $S_{ab}$ and the acceleration ${\dot{u}}^a$. Similarly, Eq. (5) corresponds to

36 $$\begin{aligned} \left( {h_i}^c \, {h_j}^d - \frac{h_{ij}}{3} \, h^{cd} \right) S_{cd} = -2\eta \left( \nabla _{(i} u_{j)} -\frac{\nabla _c u^c}{3} \, h_{ij} \right) ,\nonumber \\ \end{aligned}$$

which is impossible to satisfy in general if $S_{ab}$ does not have a special relation with $u^a$ and ${\dot{u}}^a$ as it happens instead in scalar-tensor gravity, where $u^c$ is the (normalized) gradient of the gravitational scalar degree of freedom $\phi$ and $S_{ab}=T_{ab}^{(\phi )}$ is built out of $\phi$ and its derivatives.

### Examples

As the first example of the imperfect fluid decomposition of a symmetric tensor, consider the metric itself, $S_{ab}=g_{ab}$ (“imperfect fluid” decomposition is just a name here since the dimensions of $g_{ab}$ are not those of a stress-energy tensor). The formal imperfect fluid quantities are

37 $$\begin{aligned} \rho ^\mathrm {(g)}= & {} g_{ab} u^a u^b =-1 , \end{aligned}$$

38 $$\begin{aligned} P^\mathrm {(g)}= & {} \frac{1}{3} \, h^{ab} g_{ab} = 1 ,\end{aligned}$$

39 $$\begin{aligned} q_a^\mathrm {(g)}= & {} -{h_a}^c \, g_{cd} u^d = -{h_a}^c \, u_c =0 ,\end{aligned}$$

40 $$\begin{aligned} \pi _{ab}^\mathrm {(g)}= & {} \left( {h_a}^c \, {h_b}^d {-}\frac{1}{3} \, h_{ab}\, h^{cd}\right) h_{cd} = h_{ad}\, {h_b}^d {-}\frac{3}{3} \, h_{ab} {=}0 . \nonumber \\ \end{aligned}$$

The corresponding imperfect “fluid” reduces to a perfect one with equation of state $P_\mathrm {(g)}= - \rho _\mathrm {(g)}$. Indeed, the cosmological constant term $\Lambda g_{ab}$ in the Einstein equations

41 $$\begin{aligned} R_{ab} -\frac{1}{2} \, g_{ab} R +\Lambda g_{ab} =8\pi G \, T_{ab}^\mathrm {(matter)} \end{aligned}$$

can be seen as an effective fluid with stress-energy tensor $T_{ab}^{(\Lambda )} =-\frac{ \Lambda }{8\pi G} \, g_{ab}$ and with the properties above. In addition, the constants $\Lambda$ and *G* in $S_{ab}=-\frac{\Lambda }{8\pi G} \, g_{ab}$ give this tensor the correct dimensions for a stress-energy tensor.

As a second example consider the Ricci tensor, $S_{ab} = R_{ab}$. The effective dissipative fluid quantities are related to the components of $R_{ab}$ in the frame of the observers with 4-velocity $u^a$:

42 $$\begin{aligned} \rho ^\mathrm {(Ricci)}= & {} R_{ab} u^a u^b =R_{00} , \end{aligned}$$

43 $$\begin{aligned} P^\mathrm {(Ricci)}= & {} \frac{1}{3} \, h^{ab} R_{ab} ,\end{aligned}$$

44 $$\begin{aligned} q_i^\mathrm {(Ricci)}= & {} - {h_i}^c R_{cd} u^d= - R_{i0} ,\end{aligned}$$

45 $$\begin{aligned} \pi _{ij}^\mathrm {(Ricci)}= & {} \left( {h_i}^c \, {h_j}^d - \frac{h_{ij}}{3} \, h^{cd} \right) R_{cd} = R_{ij}- \frac{ h^{cd}R_{cd}}{3} \, h_{ij} ,\nonumber \\ \end{aligned}$$

where $$i,j=1,2,3$$.

Finally, any purely spatial tensor (such as the extrinsic curvature $K_{ij}$, the shear tensor $\sigma _{ij}$, or the 3-metric $h_{ij}$ itself) will have vanishing effective $\rho$ and $q_a$ and non-vanishing effective “stresses” (including *P* and $\sigma _{ab}$).

## Conclusions

The correct Eckart generalization of the Fourier law is important for the study of the first-order thermodynamics of scalar-tensor gravity in cosmology, which is now made completely legitimate by our considerations of Sects. 2 and 3. The discussion has been extended to include spatially homogeneous and anisotropic Bianchi universes, not discussed before. The analysis of specific Bianchi models with regard to the general thermodynamical ideas advanced in previous publications involves phase space analyses and much detail and will be pursued elsewhere.

A key point of the first-order thermodynamics of scalar-tensor gravity is often misunderstood and has not been spelled out thus far. Writing the field equations of scalar-tensor gravity as effective Einstein equations produces an effective stress-energy tensor $T_{ab}^{(\phi )}$ as a source. The latter has the form (2) of an imperfect fluid energy-momentum tensor, but this fact contains no physics: *any* symmetric two-index tensor admits this decomposition, which is purely mathematical. It is the almost miracolous fact that the effective $\phi$-fluid quantities thus derived satisfy Eckart’s constitutive relations (which, in non-relativistic physics, characterize a Newtonian fluid) that make the first-order thermodynamics work.

## Data Availability

This manuscript has no associated data or the data will not be deposited. [Authors’ comment: There are no data associated with this article because of its theoretical and mathematical nature.]

## Appendix A: Force parallel to a worldline

When a 4-force parallel to the wordline of a particle (i.e., to its 4-tangent) is present, the equation of motion of this particle coincides with the non-affinely parametrized geodesic equation. Let $\tau$ be the *proper time* along this worldline (not an affine parameter) and let

A.1 $$\begin{aligned} u^c \equiv \frac{dx^c}{d\tau } , \quad \quad a^c \equiv \frac{d^2 x^c}{d\tau ^2} = \frac{du^c}{d\tau } \end{aligned}$$

according to the standard definitions of 4-velocity and 4-acceleration. In cosmology, the comoving time *t* is the proper time of comoving observers but is not an affine parameter along their worldlines unless the cosmic fluid is a dust or a cosmological constant term because these observers are accelerated by a pressure gradient pointing in the direction of comoving time. Of course, one can always introduce an affine parameter along these fluid worldlines, but this is not convenient since one wants instead to use formulas written in comoving coordinates, associated with the physical observers seeing the cosmic microwave background homogeneous and isotropic around them (on average).

Let *s* be an affine parameter along the cosmic fluid worldlines. We have

A.2 $$\begin{aligned} u^c\equiv & {} \frac{dx^c}{d\tau } = \frac{dx^c}{ds} \, \frac{ds}{d\tau } , \end{aligned}$$

A.3 $$\begin{aligned} a^c\equiv & {} \frac{d^2x^c}{d\tau ^2} = \frac{du^c}{d\tau } = \frac{d}{d\tau } \left( \frac{dx^c}{ds} \, \frac{ds}{d\tau } \right) \end{aligned}$$

A.4 $$\begin{aligned}= & {} \frac{d^2 x^c}{d\tau \,ds} \, \frac{ds}{d\tau } + \frac{d x^c}{ds} \, \frac{d^2s}{d\tau ^2}\nonumber \\= & {} \left[ \frac{d}{d\tau } \left( \frac{dx^c}{ds} \right) \right] \frac{ds}{d\tau } + \frac{dx^c}{d\tau } \, \frac{d\tau }{ds} \, \frac{d^2s}{d\tau ^2} \nonumber \\= & {} \frac{ds}{d\tau } \left[ \frac{d}{ds} \left( \frac{dx^c}{ds}\right) \right] \frac{ds}{d\tau } + u^c \, \frac{d\tau }{ds}\, \frac{d^2 s}{d\tau ^2} \end{aligned}$$

and

A.5 $$\begin{aligned} a^c = \frac{d^2 x^c}{ds^2} \left( \frac{ds}{d\tau } \right) ^2 + u^c \, \frac{d\tau }{ds} \, \frac{d^2 s}{d\tau ^2} , \end{aligned}$$

or

A.6 $$\begin{aligned} \frac{d^2 x^c}{ds^2} = a^c \left( \frac{d\tau }{ds} \right) ^2 -u^c \left( \frac{d\tau }{ds} \right) ^3 \frac{d^2s}{d\tau ^2} . \end{aligned}$$

Now, since *s* is an affine parameter along the wordline,

A.7 $$\begin{aligned} \frac{d^2 x^c}{ds^2} + \Gamma ^c_{ab} \, \frac{dx^a}{ds} \, \frac{dx^b}{ds} =0 , \end{aligned}$$

or

A.8 $$\begin{aligned} a^c \left( \frac{d\tau }{ds} \right) ^2 - u^c \left( \frac{d\tau }{ds}\right) ^3 \frac{d^2s}{d\tau ^2} +\Gamma ^c_{ab} \, \frac{dx^a}{d\tau } \, \frac{dx^b}{d\tau } \left( \frac{d\tau }{ds} \right) ^2 =0\nonumber \\ \end{aligned}$$

and

A.9 $$\begin{aligned} a^c +\Gamma ^c_{ab} \, \frac{dx^a}{d\tau } \, \frac{dx^b}{d\tau } = u^c \, \frac{d\tau }{ds} \, \frac{d^2s}{d\tau ^2} . \end{aligned}$$

We also have

A.10 $$\begin{aligned} \frac{d^2\tau }{ds^2}= & {} \frac{d}{ds} \left( \frac{1}{ds/d\tau } \right) = \frac{d\tau }{ds} \frac{d}{d\tau } \left( \frac{1}{ds/d\tau } \right) \nonumber \\= & {} -\frac{d\tau }{ds} \, \frac{ d^2s/d\tau ^2}{\left( ds/d\tau \right) ^2} = - \left( \frac{d\tau }{ds} \right) ^3 \frac{ d^2s}{d\tau ^2} , \end{aligned}$$

then Eq. (A.9) can be written also as

A.11 $$\begin{aligned} a^c +\Gamma ^c_{ab} \, \frac{dx^a}{d\tau } \, \frac{dx^b}{d\tau } = - u^c \left( \frac{d\tau }{ds} \right) ^{-2} \frac{d^2 \tau }{d s^2} . \end{aligned}$$

The orthogonality of the 4-acceleration $a^c$ to the 4-velocity engraved in the mind of relativists, $a^c u_c=0$, follows from differentiating the normalization $u^c u_c=-1$, but $dx^c/ds$ is not normalized and $g_{ab} \, \frac{d^2x^a}{ds^2} \, \frac{dx^b}{ds} \ne 0$. In fact,

A.12 $$\begin{aligned} g_{ab} \, \frac{d^2x^a}{ds^2} \, \frac{dx^b}{ds}= & {} g_{ab} \left( a^a {-}u^a \, \frac{d\tau }{ds} \, \frac{d^2s}{d\tau ^2} \right) \frac{ 1}{ \left( ds/d\tau \right) ^2} \frac{ dx^b}{d\tau } \, \frac{d\tau }{ds} \nonumber \\= & {} - g_{ab} u^a u^b \, \frac{d^2s}{d\tau ^2} \left( \frac{d\tau }{ds} \right) ^3 \frac{d\tau }{ds} \nonumber \\= & {} \left( \frac{d\tau }{ds} \right) ^4 \frac{d^2s}{d\tau ^2} , \end{aligned}$$

which is different from zero unless $\tau$ is already an affine parameter. Likewise, we have

A.13 $$\begin{aligned} g_{ab} \, \frac{dx^a}{ds} \, \frac{dx^b}{ds} = g_{ab} \, \frac{dx^a}{d\tau } \, \frac{dx^b}{d\tau } \, \left( \frac{d\tau }{ds} \right) ^2 {=}{-}\left( \frac{d\tau }{ds} \right) ^2 \ne {-}1.\nonumber \\ \end{aligned}$$

## Footnotes

1 The construction of an effective $T_{ab}$ extends to Nordström gravity, which is not a scalar-tensor but a purely scalar theory with less degrees of freedom than GR, and yields a negative temperature with respect to GR [68].

2 Whether this 4-force parallel to the trajectory can legitimately be called a “force” is a matter of semantics. Similarly, the 4-acceleration ${\dot{u}}^a$ of a particle is often taken to be synonymous of its spatial projection $h_{ab} {\dot{u}}^b$. For clarity, here we make the distinction explicit.

3 Interestingly, this correct form appears in Eckart’s original discussion [64] in which, however, there is no mention of the possibility of 4-accelerations parallel to 4-velocities. Indeed, Eckart’s work [64] predates all the literature on such instances [56, 74–83, 86–92].

4 An observer in a frame moving with respect to $u^a$ would see this effective fluid tilted and would experience a non-vanishing energy flux $q_a '$ which is, however, purely convective [96, 97].

5 See [102] for an attempt to generalize the analogy to a non-Newtonian fluid with alternative constitutive relations non-linear in the gradient of the fluid 4-velocity.
